# 1047. Development of a Next Generation 30^+^ Valent Pneumococcal Conjugate Vaccine (VAX-XP) Using Site-Specific Carrier Protein Conjugation

**DOI:** 10.1093/ofid/ofab466.1241

**Published:** 2021-12-04

**Authors:** Chris Behrens, Jeff Fairman, Paresh Agarwal, Shylaja Arulkumar, Sandrine Barbanel, Leslie Bautista, Aym Berges, John Burky, Peter Davey, Chris Grainger, Sherry Guo, Sam Iki, Mark Iverson, Neeraj Kapoor, Olivier Marcq, Thi-Sau Migone, Lucy Pill, Mohammed Sardar, Paul Sauer, James Wassil

**Affiliations:** Vaxcyte, Inc, Foster City, California

## Abstract

**Background:**

Due to the diversity of serotypes, exacerbated by the phenomenon of serotype replacement, there remains an unmet medical need for a pneumococcal conjugate vaccine (PCV) containing additional serotypes. Using a cell-free protein synthesis (CFPS) platform to produce an enhanced carrier protein (eCRM^®^) based on the CRM_197_ sequence, Vaxcyte is developing a PCV encompassing over 30 serotypes. The eCRM carrier protein contains multiple insertions of the non-native amino acid para-azidomethyl-L-phenylalanine (pAMF) that facilitates site-specific conjugation of the pneumococcal polysaccharides (PS) to eCRM. Unlike conventional methodologies, site-selective conjugation enhances process consistency and increases capacity for inclusion of additional serotypes in a PCV without promoting carrier suppression. Using this platform, the aim of the current study was to employ CFPS technology to construct a 31-valent PCV and evaluate its immunogenicity in New Zealand White (NZW) rabbits.

**Methods:**

The eCRM carrier protein was individually conjugated to each of 31 selected pneumococcal PSs using copper-free click chemistry to produce 31 Conjugate Drug Substances (DS), which were then mixed with aluminum phosphate to produce the VAX-XP Drug Product. 24 of the DS conjugates in VAX-XP were generated at manufacturing scale. Two doses of VAX-XP were administered to NZW rabbits at 0 and 21 days to assess its ability to elicit anti-capsular IgG antibodies. Additionally, rabbits were also administered either Prevnar13 or a mixture of Pneumovax 23 and 8 incremental PS in isotonic saline, as comparators.

**Results:**

VAX-XP showed conjugate-like immune responses for all 31 serotypes, as demonstrated by superior responses to PS-based vaccines and comparable responses to Prevnar13. IgG responses for VAX-XP compared with Prevnar13 and Pneumovax 23 at 14 days post dose 2

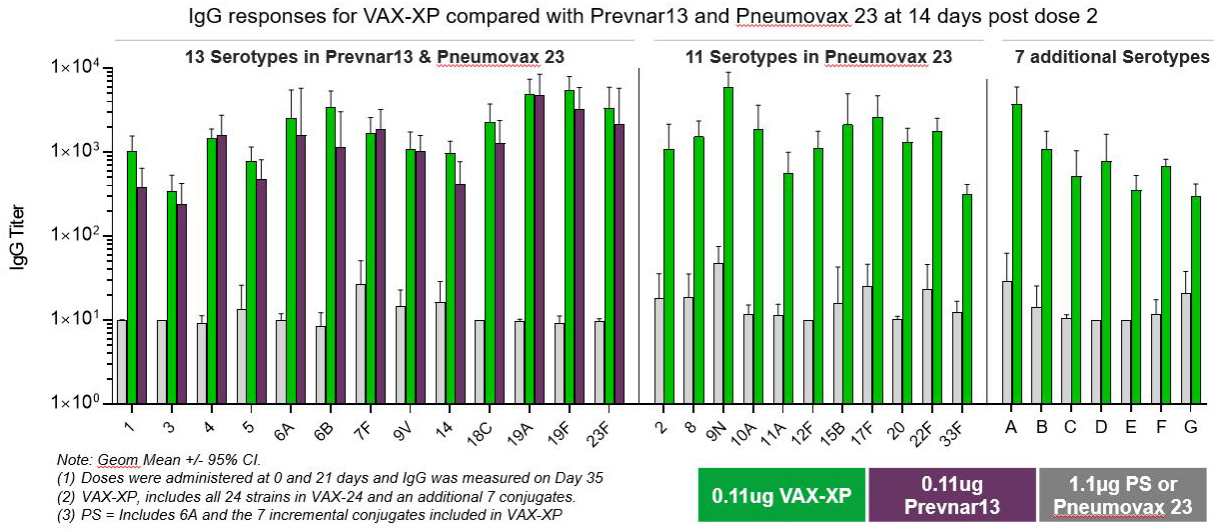

**Conclusion:**

These results demonstrate that increasing the number of pneumococcal serotypes does not result in immunological attenuation in any of the serotypes contained in VAX-XP relative to the current standard of care. Furthermore, the data confirm the scalability and reproducibility of the CFPS platform in the production of VAX-XP conjugates, creating the foundation for a next generation broad-valency PCV.

**Disclosures:**

**Chris Behrens, PhD**, **Vaxcyte, Inc.** (Employee) **Jeff Fairman, PhD**, **Vaxcyte, Inc.** (Employee) **Paresh Agarwal, PhD**, **Vaxcyte, Inc.** (Employee) **Shylaja Arulkumar, MS**, **Vaxcyte, Inc.** (Employee) **Sandrine Barbanel, MS**, **Vaxcyte, Inc.** (Employee) **Leslie Bautista, n/a**, **Vaxcyte, Inc.** (Employee) **Aym Berges, PhD**, **Vaxcyte, Inc.** (Employee) **John Burky, BS**, **Vaxcyte, Inc.** (Employee) **Peter Davey, MS**, **Vaxcyte, Inc.** (Employee) **Chris Grainger, PhD**, **Vaxcyte, Inc.** (Employee) **Sherry Guo, PhD**, **Vaxcyte, Inc.** (Employee) **Sam Iki, MS**, **Vaxcyte, Inc.** (Employee) **Mark Iverson, BS**, **Vaxcyte, Inc.** (Employee) **Neeraj Kapoor, PhD**, **Vaxcyte, Inc.** (Employee) **Olivier Marcq, PhD**, **Vaxcyte, Inc.** (Employee) **Thi-Sau Migone, PhD**, **Vaxcyte, Inc.** (Employee) **Lucy Pill, MS**, **Vaxcyte, Inc.** (Employee) **Mohammed Sardar, n/a**, **Vaxcyte, Inc.** (Employee) **Paul Sauer, MBA**, **Vaxcyte, Inc.** (Employee) **James Wassil, MS MBA**, **Vaxcyte, Inc.** (Employee)

